# Information-Driven Active Audio-Visual Source Localization

**DOI:** 10.1371/journal.pone.0137057

**Published:** 2015-09-01

**Authors:** Niclas Schult, Thomas Reineking, Thorsten Kluss, Christoph Zetzsche

**Affiliations:** Cognitive Neuroinformatics, Bremen University, Bremen, Germany; Peking University, CHINA

## Abstract

We present a system for sensorimotor audio-visual source localization on a mobile robot. We utilize a particle filter for the combination of audio-visual information and for the temporal integration of consecutive measurements. Although the system only measures the current direction of the source, the position of the source can be estimated because the robot is able to move and can therefore obtain measurements from different directions. These actions by the robot successively reduce uncertainty about the source’s position. An information gain mechanism is used for selecting the most informative actions in order to minimize the number of actions required to achieve accurate and precise position estimates in azimuth and distance. We show that this mechanism is an efficient solution to the action selection problem for source localization, and that it is able to produce precise position estimates despite simplified unisensory preprocessing. Because of the robot’s mobility, this approach is suitable for use in complex and cluttered environments. We present qualitative and quantitative results of the system’s performance and discuss possible areas of application.

## Introduction

One of the guiding principles in modern robotics is to mimic biological and in particular human perceptual processes. Human perception is inherently multisensory, that is, every percept is a product of an integrative process of multiple sensory modalities. Consequently, in recent years, sensor fusion and multisensory research began to influence each other, linking both fields of research on a fundamental level: In multisensory research, statistically optimal solutions for the integration of data from multiple sensory modalities have become an important approach for understanding multisensory integration processes of basic features [1] [2] [3] and sensory coding in general [4]. Statistical optimality has always been a dominant approach in the field for sensor fusion because it provides an inherent link to information theory, which is the mathematically intuitive approach for the combination of noisy data originating from multiple sensors.

Source detection and localization received a lot of attention in the last decade and there are a variety of unisensory [5] [6] [7] as well as multisensory [8] [9] [10] approaches that have been proposed. While there are approaches for accurate and relatively robust *auditory* source localization, they often require expensive microphone arrays [7] [11] [12] [13] and are not suited for biologically plausible modeling. *Auditory* source localization systems also tend to be prone to noise and reverberation, so that an additional source of information is highly advisable in order to increase robustness of the system. In contrast, unisensory systems utilizing only *visual* input are problematic, because most cameras feature only a small field of view, limiting their usefulness for source detection and localization systems. Moreover, ambiguous scenes and occlusion can degrade performance. Consequently, utilizing multiple sensory modalities, in particular combining the complementary properties of vision and audition, seems to be a promising approach to solve these problems: Auditory source localization systems have the advantage that they can monitor all directions, allowing a quick disambiguation of the scene while the visual modality offers a high spatial resolution, which allows a system to improve the rough audition-based estimate as soon as the set of possible locations is sufficiently small. Audio-visual approaches to source localization for use on mobile robots [14] [15] are rare and mostly focused on specific areas of application, where the movements of the robot were unrelated to the source localization system. In contrast, our system incorporates autonomous movements of the robot to optimize source localization performance.

As an alternative to existing systems, we present a biologically-inspired audio-visual approach to the problem of source localization using stereo microphones and a camera. The main novelty of our approach is twofold: First, we use a mobile robot that integrates measurements obtained from different locations using a particle filter. Second, we use an information gain (IG) mechanism for selecting the most effective actions in order to actively reduce uncertainty via movements. The system accomplishes this selection by analyzing its current belief distribution about the source location and by determining a set of possible actions. It then predicts the influence of each of these actions and the consecutive auditory and visual measurements at the respective target position on its current belief distribution. After comparing the results of these predictions, the system selects the most informative action in order to minimize the expected uncertainty. This approach is inspired by the recent development of theories which reject the assumption of perception as a mostly passive generalization of the properties of coincident stimuli. In particular, the development of theories of the embodied mind in empirical psychology and philosophy of nature [16], the proposal of sensorimotor systems in biologically-inspired research in computer science [17], the reinterpretation of the role of motor actions as the binding element between the senses in multisensory research [18], as well as active perception approaches in robotics [19] all suggest that multisensory perception is an inherently active process. In practice, possible fields of application of the proposed system include social and rescue robotics as well as automatic camera control systems for video conferences.

## Methods

**Fig 1:**
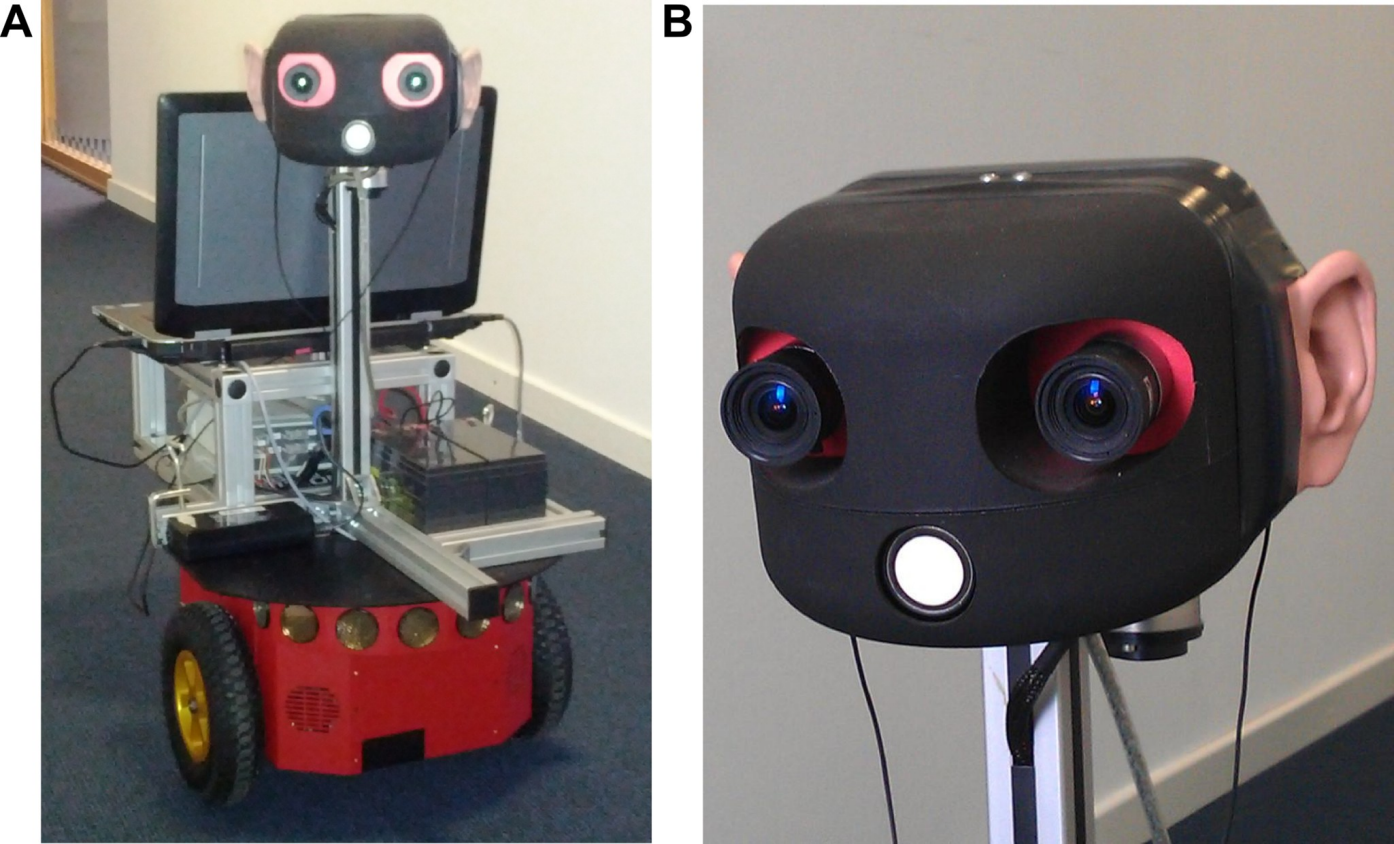
Robot and Robohead. A) The robot we use for evaluating the proposed approach. B) The robot is equipped with a rotatable head, which features an integrated camera, biologically-realistic pinnae, and in-ear stereo microphones.

We implemented the system on a mobile robot (Pioneer P3-DX), on which we mounted a robot head which can perform -90 to +90 degrees rotations in the azimuth plane and -30 to +30 degrees rotations in the median plane. The robot’s head features an integrated camera system as well as in-ear stereo microphones, which are mounted in biologically-realistic human-like pinnae (Kemar KB0065/66) attached to the sides of the robot’s head (see Fig 1). This setup is designed to mimic the human outer ear system (that is, pinna, auditory canal and eardrum) in order to use a biologically realistic setup and to allow basic modeling of human auditory processes.

**Fig 2:**
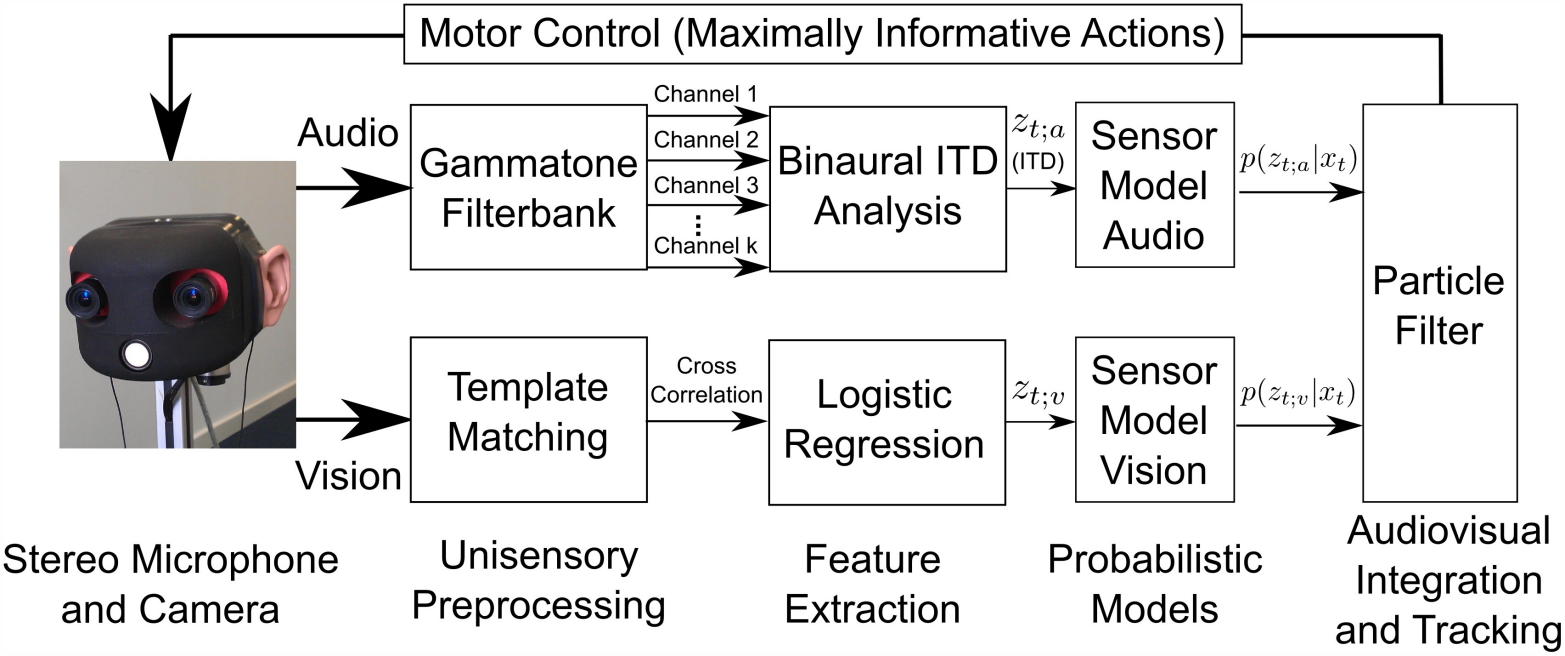
System Overview. The main components and basic dataflow of the proposed information-driven multisensory system.

An overview of the system’s main components and the basic dataflow is shown in Fig 2. After each action audio data and camera images are recorded. Currently, the system only processes measurements between the execution of actions in order to avoid noisy measurements resulting from motor sounds and moving sensors. The recorded stereo audio data is then transformed into a time-frequency representation, which is used for sound source localization by estimating interaural temporal differences (ITDs). The recorded camera images are evaluated by applying an object detection algorithm and by assigning probabilities for the presence of the source to each of the positions of possible targets inside the image. The integration of auditory and visual measurements as well as the temporal integration of consecutive measurements is achieved by a particle filter, which updates the current belief distribution over possible source positions based on a sensor model for each modality linking the respective measurements to the associated states.

A key feature of the architecture proposed in this paper is the utilization of an IG mechanism for the selection of the optimal action in each step, which is indicated as a feedback loop in Fig 2. Based on the belief distribution about the source’s current position, the system predicts the effects of a set of possible actions, estimates their expected influence on the belief distribution and executes the most informative action. This is repeated until the uncertainty is smaller than a threshold. The components of the system are described in greater detail in the following subsections.

### Audio Processing

After each movement, audio data is recorded by the pinna-modulated stereo microphones and the recorded data of both channels is transformed into a biologically-plausible time-frequency representation by a gammatone filterbank [20].

The basic time-domain filter equation (the impulse response) of Gammatone filters is defined as

$$\begin{array}{c} g\left(t\right)=at^{n-1}e^{-2\pi bt}cos\left(2\pi ft+\varphi \right). \end{array}$$ (1)

Here, *a* denotes the amplitude, *t* denotes time, *n* denotes the order of the filter, *b* the bandwidth, *f* the center frequency, and *φ* the phase of the filter. The impulse responses of Gammatone filters are effectively a product of a tone (cosine) and a scaled gamma function. The resulting time-frequency representation is commonly known as a cochleagram, implying that the resulting time-frequency representation is trying to model the processing properties of the human cochlea. In the following, we denote the cochleagrams for the left and the right ear as **c**_*l*_[*k*, *t*] = **a**_*l*_[*t*] ∗ *g*_*k*_ and **c**_*r*_[*k*, *t*] = **a**_*r*_[*t*] ∗ *g*_*k*_, where the asterisk denotes filtering, *g*_*k*_ denotes the *k*-th filter in the filterbank and **a**_*l*_[*t*] and **a**_*r*_[*t*] represent the auditory input of the left and right ear, respectively.

We choose Gammatone filterbanks as a means for generating a time-frequency representation because they are specifically designed to provide a biologically-plausible but still computationally-efficient solution. Since their introduction in [20], they have become the standard time-frequency-representation in auditory modeling for real-time systems [21]. With appropriate parameterization (based on empirical biological data, typically the ERB-scale, see [22]), the transfer functions of Gammatone filters are close to those of human inner-ear cells, showing, e.g., the characteristic increase of bandwidth with increasing center frequency of the filters. There are efficient implementations of Gammatone filters available, utilizing complex recursive time domain filters [23], which is desirable for use on a robot.

**Fig 3:**
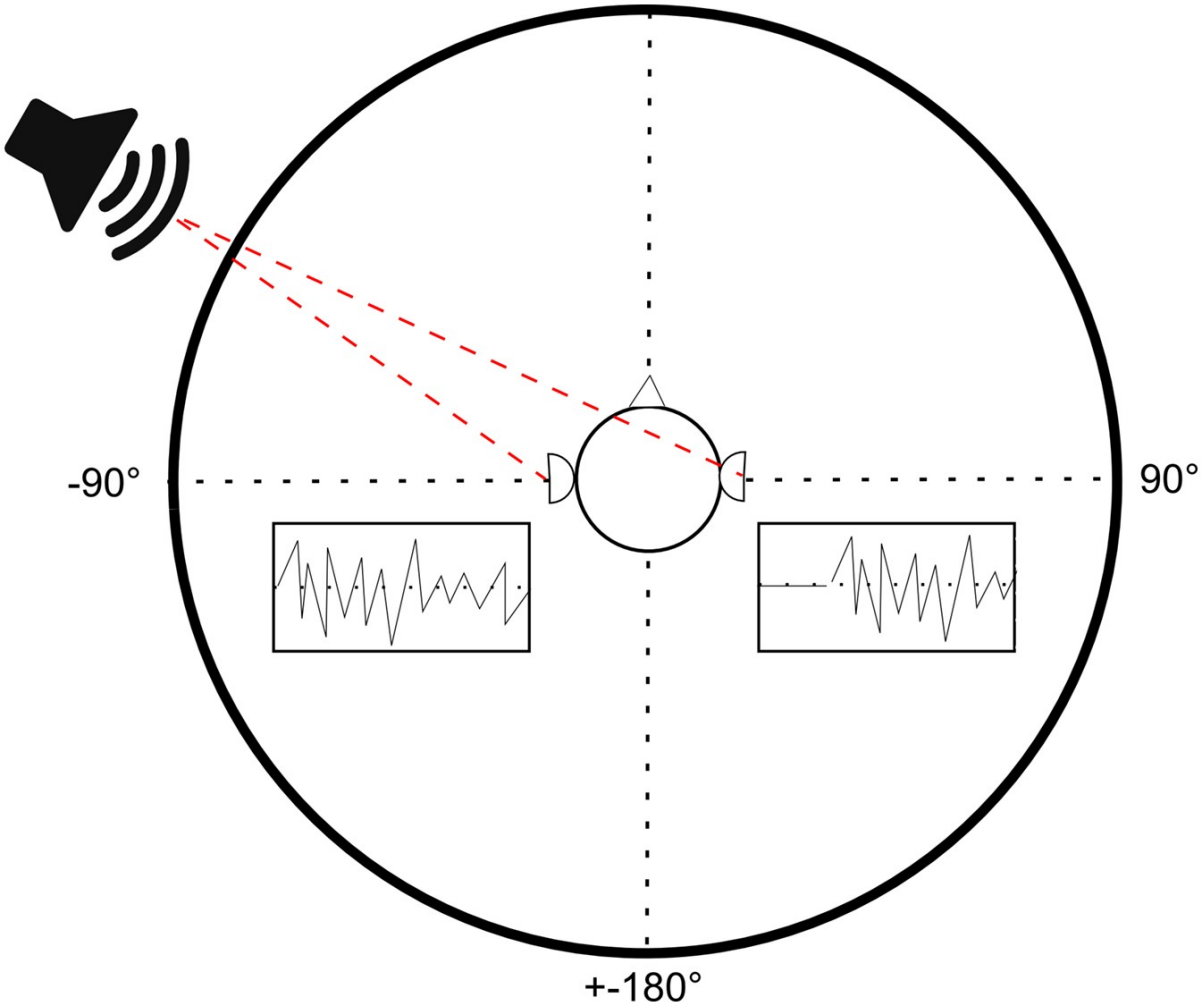
Interaural Temporal Differences. Depending on the position of the source, the emitted sound needs a different amount of time to travel to the individual ears. By estimating this interaural delay, the listener is able to identify the position of the source.

We use a filterbank with 128 channels with center frequencies between 75 and 1800 Hz. After generating cochleagrams for the audio input of the left and right ears, these are used to estimate the position of the source. For auditory source localization we use a classic binaural approach based on ITDs [24](While a filterbank with 128 channels seems excessive for the calculation of ITDs, we choose to include a high number of channels because we are interested in investigating whether the transfer function induced by the artificial pinnae influences the performance of ITD-based source localization.): The basic idea of this approach is that sound takes time to travel from one ear to the other and that this temporal delay can be utilized to determine the azimuthal position of the source. This delay increases monotonically with the azimuth angle, though not linearly (at least in our case; it depends on the shape of the robot’s head). The basic principle is illustrated in Fig 3. In practice, we measure the difference of the time of arrival between the left and right channel. In our system, this is achieved by calculating the normalized cross-correlation between the cochleagram representations of the recorded audio signals of the left and the right ear

$$\begin{array}{c} R\left[t\right]=\frac{\sum_{k^{\prime },t^{\prime }}\left(c_{l}\left[k^{\prime },t^{\prime }\right]c_{r}\left[k+k^{\prime },t+t^{\prime }\right]\right)}{\sqrt{\sum_{k^{\prime },t^{\prime }}c_{l}{\left[k^{\prime },t^{\prime }\right]}^{2}\sum_{k^{\prime },t^{\prime }}c_{r}{\left[k+k^{\prime },t+t^{\prime }\right]}^{2}}}. \end{array}$$ (2)

Here, **c**_*l*_ and **c**_*r*_ denote the left and right cochleagram, respectively, *k* denotes the channel within the filterbank and *t* denotes time. The correlation results can be used to estimate the temporal delay between the left and right ear by identifying the index *t* for which cross-correlation is maximal. The measured delays with maximum correlation can then be mapped to their corresponding angles because this mapping is monotonous and relatively unambiguous. In order to estimate a reasonably precise mapping, we measure ITDs for the robot’s head with approximately 6 degree spacing using a speaker array arranged in a semi-circle in a semi-anechoic chamber for 240 training samples. For each of the 31 positions we then calculate the mean ITD (averaged over all training samples for the respective position) and the corresponding standard deviation to define our auditory sensor model, which is used to find the angles corresponding to a particular delay and is described in further detail below. The decision to calculate ITDs utilizing a cochleagram representation (in contrast to calculate them directly on the time-domain data) is mostly motivated by the observation that time-frequency representations seem to be less prone to noise. Furthermore, as we are using bandpass filters with center frequencies between 75 and 1800 Hz, only a relatively limited set of frequency bands is considered, which reduces sensitivity to high-frequency noise.

On a test set of 480 audio files (15 for each speaker-position) recorded in the same semi-anechoic chamber, this mapping results in perfect performance (i.e., correct identification of speaker position), while noisy and reverberant audio data lead to decreases in performance and in some rare cases even to wrong estimates. Thus, in noisy or reverberant rooms, preprocessing with denoising and dereverberation algorithms is advisable. We intentionally choose to only use ITDs as auditory features in order to show that even a simplified auditory feature extraction process is sufficient to get precise position estimates when utilizing it on a mobile system. But it is important to note that there are restrictions to utilizing ITDs for auditory localization: Most importantly, no localization in median plane is possible because, in most cases, there are no position-dependent temporal delays between the ears which would allow the robot to differentiate elevations. Furthermore, ITDs are not suitable for frequency bands over approx. 1600 Hz because the short wavelength of these sound waves leads to phase ambiguities [25] [26]. However, Breebart et al showed for human listeners that low-frequency HRTF phase characteristics can be described by a single interaural delay without significant decrease of localization performance and that the absence of high-frequency phase information does not result in perceivable changes [27]. Thus, as long as we are focusing on azimuthal localization, utilizing only ITDs based on low-frequency cochleagram-data seems to be a reasonable approximation. It should be noted that, in order extend our approach to localization in the median plane, it would be necessary to modify the auditory feature extraction stage to be able to utilize frequency-dependent interaural level differences (ILDs) or explicit HRTF-based cues, which inherently combine both features (ITDs and ILDs) [28] [29].

Using only ITDs for auditory source localization has the further disadvantage, that the estimation of a source’s distance is principally impossible, because ITDs are not distance-dependent, but we show below that our system is able to overcome this restriction by performing actions during source localization.

### Visual Processing

In each step, images are recorded by the robot’s camera and an object detection algorithm is applied to the recorded image in order to determine the position of the source. We use a simple template matching approach for the detection of objects, again utilizing normalized cross-correlation defined in Eq (2) as a similarity measure. We use multiple templates for each object in order to achieve basic rotation- and scale-invariance. We choose an object detection approach based on template matching utilizing a filterbank and normalized cross-correlation in order to introduce a basic parallelism between the auditory and the visual modality to the system, which is indicated by biological and psychological research. Moreover, as we mostly want to evaluate the performance of the information-driven action-selection process, we use a rather simple approach for visual source localization.

Before applying the template matching algorithm, all camera images and all templates are filtered by a first-order Gaussian derivative filter (with standard deviation 1.8) in x- and in y-direction. This filtering is a combination of a smoothing (Gaussian lowpass) and a gradient operation (derivative), which reduces the system’s susceptibility to noise and avoids some of the problems typical for template-matching based approaches (like issues with areas of uniform intensity and contrast inconsistencies due to illumination changes). It also introduces basic invariance properties to the system: The smoothing operation leads to a greater robustness with respect to rotation and scale while the gradient operation reduces the effects of illumination and contrast variations.

The resulting cross-correlation-images for the different templates are combined by a maximum-operation

$$\begin{array}{c} I_{max}\left[x,y\right]=max\left(I_{0}\left[x,y\right],I_{1}\left[x,y\right],...,I_{n}\left[x,y\right]\right), \end{array}$$

where *I*_*k*_ denotes the “cross-correlation-image” for the *k*-th template. Consequently, we analyze each of the cross-correlation images and determine the maximum correlation for each pixel position. Since we are currently only interested in localization in the azimuth plane, we also apply a maximum-operation over all elevations:

$$\begin{array}{c} z_{v}\left[x\right]=max_{y}\left(I_{max}\left[x,y\right]\right). \end{array}$$

This operation results in a one-dimensional feature vector *z*_*v*_, whose individual elements correspond to the maximum correlation for a particular azimuthal position. Below we explain how these feature vectors are used for source localization in more detail.

A problem of utilizing the visual modality for source detection and localization is that, in many cases, the source is not visible in the camera image due to a relatively small field-of-view. Especially during the early phases of source detection (that is, when the system has very little information about the source’s position), the usefulness of the visual modality for source localization is therefore limited. In contrast, as soon as the system has a rough estimate, it can utilize vision to improve the spatial accuracy.

In order to calculate probabilities for the presence of the source for each pixel/direction in the recorded image based on the template matching results, we use logistic regression [30] [31]. To train the logistic regression model, we use a set of training images recorded by the robot’s camera under realistic conditions, apply the template matching procedure and manually label the positions of the respective bounding boxes of the source. This means that all locations/pixel positions which are occupied by the source are labeled with 1 while empty/wrong positions are labeled with 0. This allows us to utilize the resulting Boolean “bounding box images” to train the logistic regression model, which we can use to convert the template matching results to probabilities for the presence of the source.

**Fig 4:**
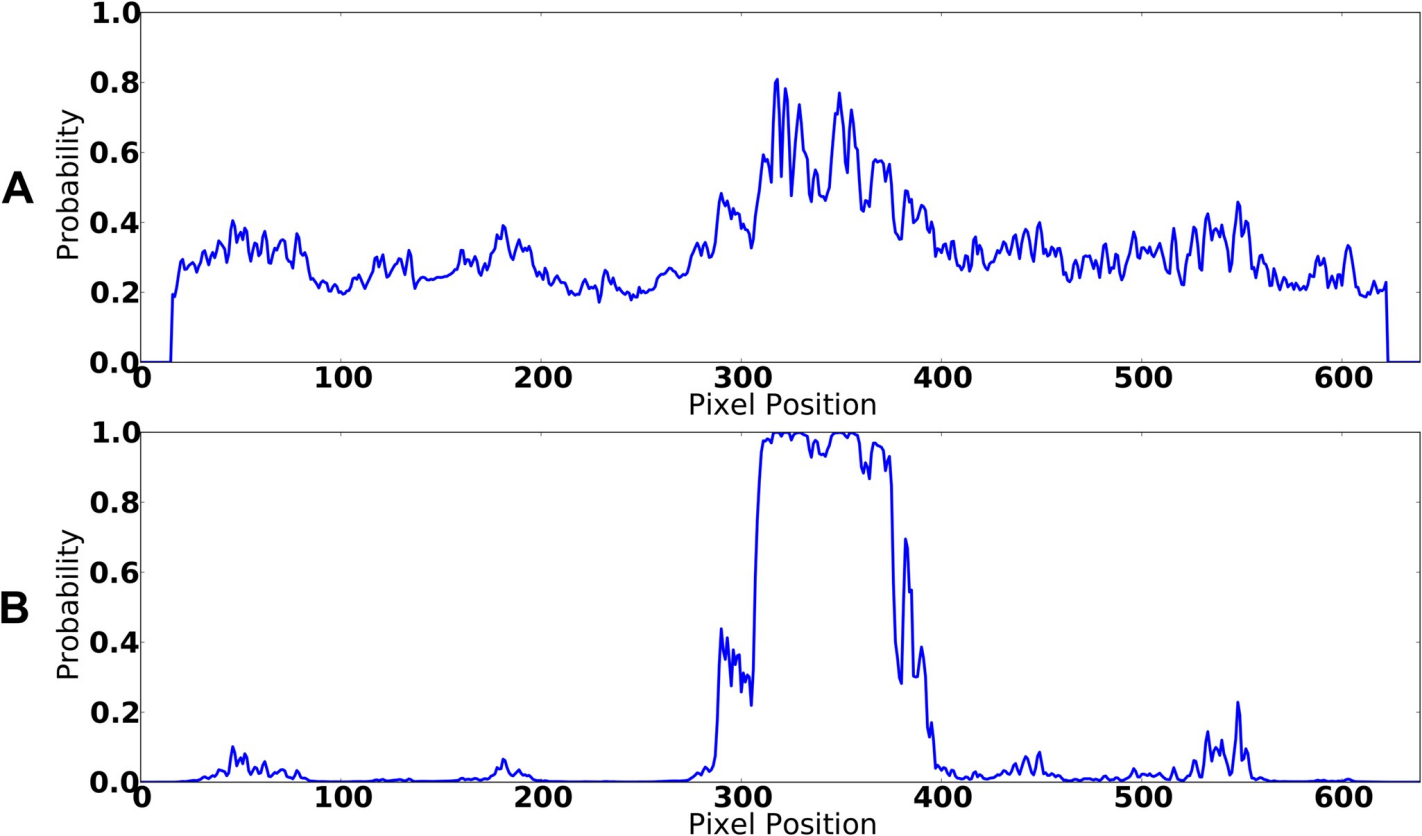
Visual Processing: Logistic Regression. A) Result of the template matching procedure B) Effect of the application of the logistic regression model.

A potential drawback of utilizing template matching for object detection are the sparse responses associated with these algorithms: They typically result in high responses for a specific location where an appropriate template is perfectly aligned, but produce low responses in the neighborhood [32]. While the mere presence of an object can be reliably detected by template matching, we do not get any appropriate spatially extended bounding boxes. In our system, this could lead to low probabilities for positions which are occupied by the source, but which are not appropriately aligned with any of the templates. This can result in low performance, because it leads to noisy data for the visual modality. Furthermore, in our implementation, template matching results already tend to be very noisy (i.e., high correlations for “wrong” locations) due to the maximum-operation over all elevations and all templates. We try to overcome these problems by not only using the correlation response for a single pixel as input for logistic regression, but also the average response of the region around a pixel position, which we combine into 2-dimensional feature vectors that are used to train the logistic regression model. Example responses of visual processing results with and without application of the logistic regression model are shown in Fig 4A and 4B. Fig 4A shows the template matching result (after the maximum-operation over all elevations and templates) and Fig 4B shows the output signal after applying logistic regression: While it is clearly visible that there is a single position which exhibits a maximal response corresponding to the most appropriate template, the application of the logistic regression model leads to increased responses inside the neighborhood of the respective pixel position and to reasonable bounding boxes for objects. Moreover, in contrast to the data generated by the template matching procedure, it results in very low probabilities for areas, in which the source is not present. This is an interesting finding, as it suggests that the application of this logistic regression model reduces the signal-to-noise-ratio of the template matching results, which deserves further investigation.

### Audio-Visual Integration

We use a particle filter based on importance resampling [33] for the integration of auditory and visual data and for the temporal integration of consecutive measurements. The basic idea of particle filters is to approximate the probability density function (PDF) of the current state by a finite set of samples (referred to as particles). While Kalman filters [34], which are the most popular approach for source localization [5] [35] [36], require the underlying models to be normally distributed, particle filters are a non-parametric approach and are thus able to represent a much larger class of distributions. In particular, particle filters allow the system dynamics and measurement update to be described by non-linear functions, and they allow the PDF of the current state to be a multi-modal distribution. Being able to represent multi-modal distributions is essential in our approach due to localization ambiguities caused by auditory measurements (see below).

The goal of audio-visual integration is to estimate the posterior PDF *p*(*x*_*t*_∣*z*_0:*t*_, *u*_0:*t*_) where *x*_*t*_ denotes the source location at time *t*, *z*_0:*t*_ = *z*_0_, …, *z*_*t*_ denotes the sequence of all measurements over time, and *u*_0:*t*_ denotes the sequence of controls the robot executes over time. The state *x*_*t*_ is a vector *x*_*t*_ = (*x*_*t*;*φ*_, *x*_*t*;*d*_, *x*_*t*;*r*_)^*T*^ where *x*_*t*;*φ*_ ∈ [−*π*, *π*] is the direction of the source and *x*_*t*;*d*_ is the Euclidean distance of the source, both expressed in a robot-centric coordinate system. In addition, *x*_*t*;*r*_ ∈ [−*π*/2, *π*/2] is the current rotation of the robot’s head with respect to the robot’s “body”, which has to be estimated as well due to noise associated with the execution of controls for head rotations. The PDF of the current state can be updated recursively over time in two steps: a prediction and a correction step. The equations underlying both steps are approximated by the particle filter [37].

#### Prediction Step

The prediction step is performed whenever the state changes. Here, we assume that the location of the source does not change over time (although this assumption is not required by our approach) and, as a result, state changes only occur due to actions by the robot. These changes are described by a dynamics model *p*(*x*_*t*_∣*x*_*t* − 1_, *u*_*t*_) where *u*_*t*_ represents an action that leads to a new state *x*_*t*_. Using the dynamics model, the state distribution *p*(*x*_*t* − 1_∣*z*_0:*t* − 1_, *u*_0:*t* − 1_) at time *t* − 1 can be updated in order to obtain the proposal distribution *p*(*x*_*t*_∣*z*_0:*t* − 1_, *u*_0:*t*_) that does not yet contain the latest measurement *z*_*t*_.

$$\begin{array}{c} p\left(x_{t}|z_{0:t-1},u_{0:t}\right)=\int_{x_{t-1}}p\left(x_{t}|x_{t-1},u_{t}\right)\;p\left(x_{t-1}|z_{0:t-1},u_{0:t-1}\right)\;dx_{t-1} \end{array}$$ (3)

The above equation holds if the underlying process satisfies the Markov property, i.e., the current state only depends on the previous one.

Because we assume a static source, the dynamics model *p*(*x*_*t*_∣*u*_*t*_, *x*_*t* − 1_) is given by simple geometric transformations describing the robot’s movement and additive Gaussian noise: The control *u*_*t*_ determines the relative 2D translation and rotation of the robot itself, and it also describes how the robot’s head rotation changes. Because the source location is represented in the robot’s coordinate system, translation and rotation of the robot simply require transforming the source location correspondingly.

For the case of a moving source, the dynamics model also has to reflect the movement of the source in addition to the coordinate system changes caused by the robot’s movement. The simplest way of achieving this would be to assume a constant velocity for the source and noisy acceleration, in which case the state vector has to be extended by the source’s velocity [38].

#### Correction Step

In the correction step, the proposal distribution produced by the prediction step is updated based on a new measurement *z*_*t*_. This is achieved by applying Bayes’ theorem where measurements are assumed to be conditionally independent.

$$\begin{array}{c} p\left(x_{t}|z_{0:t},u_{0:t}\right)\propto p\left(z_{t}|x_{t}\right)\;p\left(x_{t}|z_{0:t-1},u_{0:t}\right) \end{array}$$ (4)

The likelihood *p*(*z*_*t*_∣*x*_*t*_) represents the sensor model where measurement *z*_*t*_ = (*z*_*t*;*a*_, *z*_*t*;*v*_)^*T*^ consists of an auditory component *z*_*t*;*a*_ and a visual component *z*_*t*;*v*_. Given the current state *x*_*t*_, these components can be considered as approximately conditionally independent, which is why the likelihood in Eq (4) can be factorized into an auditory sensor model and a visual one (and in case there are additional sensors, these can be easily incorporated into the likelihood as well).

$$\begin{array}{c} p\left(z_{t}|x_{t}\right)=p\left(z_{t;a}|x_{t}\right)\;p\left(z_{t;v}|x_{t}\right) \end{array}$$ (5)

Eqs (4) and (5) provide the basis for the multisensory integration in our approach. The assumption of conditional independence in Eq (5) is quite reasonable in this context because the state (relative position of the source) determines both the visual and the auditory measurements aside from noise, which is independent for both modalities. Conditional independence only breaks down in case there are additional factors influencing both modalities that are not reflected by the state representation (e.g. a person walking between the robot and the source). However, even in such situations, the system could probably recover due to the integration of multiple measurements over time.

#### Auditory Sensor Model

The sensor model for audition *p*(*z*_*t*;*a*_∣*x*_*t*_) describes the probability density for a certain ITD given the current state *x*_*t*_. We model this distribution as a Gaussian for which we learn the parameters (mean and variance) by evaluating a test set of annotated audio files, which we recorded in a semi-anechoic chamber: Using a set of 31 speakers arranged in a semicircle (corresponding to 6 degrees separation between consecutive speakers), we present test stimuli to the robot and calculate ITDs with the procedure presented above. For each speaker position *x*_*k*_, we can then calculate the mean *μ*_*k*_ and standard deviation *σ*_*k*_ of the ITD response (*z*_*t*;*a*_), which allows us to define the sensor model for audition by:

$$\begin{array}{c} p\left(z_{t;a}|x_{t;k}\right)=\frac{1}{\sigma_{k}\sqrt{2\pi }}e^{-\frac{z_{t;a}-\mu_{k}}{2\sigma_{k}^{2}}}. \end{array}$$ (6)

In order to simulate front-back-mixups frequently found in perceptual experiments on humans in audition [39] [40], we are intentionally using the same sensor model for positions in front of the robot’s head and corresponding positions to the back of the robot’s head. As we show below, front-back-confusions can be easily disambiguated in our source localization approach by integrating multiple measurements and actions into a single estimate.

#### Visual Sensor Model

The sensor model for vision *p*(*z*_*t*;*v*_∣*x*_*t*_) directly uses the values produced by the template matching/logistic regression approach discussed above. For this, the particle location, which is represented in a robot-centric coordinate system, is first transformed into a camera-centric one and the resulting angles are mapped to their corresponding pixel positions. If the location is within the field of view of the camera, the system can now evaluate the feature vector (the output of the logistic regression model, see Fig 4) at the corresponding angle to obtain an estimate for the probability of the presence of the source for this location.

In many cases, the location corresponding to a particular direction *x*_*t*;*φ*_ is not visible in the camera image though due to the limited field of view of the camera. In these cases, the particles in question are not updated because the measurement contains no information about the corresponding locations.

### Action Selection by Information Gain Maximization

In our approach, the central principle for selecting actions is to choose the most informative ones. This is achieved by maximizing the expected information gain with respect to the current PDF approximated by the particle distribution. The expected information gain *IG* of an action *u*_*t*_ is defined as the expected difference in uncertainty (measured by entropy *H*) between the current PDF at *t* − 1 and the PDF at time *t* after having executed action *u*_*t*_ and having recorded a new measurement *z*_*t*_ [41]:

$$\begin{array}{c} IG\left(u_{t}\right)=H\left(x_{t-1}|z_{0:t-1},u_{0:t-1}\right)-E_{z_{t}}\left[H\left(x_{t}|z_{0:t},u_{0:t}\right)\right]. \end{array}$$ (7)

The distribution at time *t* − 1 is directly given because it corresponds to the current particle set. Because measurement *z*_*t*_ is not known prior to executing action *u*_*t*_, the expected value *E*_*z*_*t*__ with respect to *z*_*t*_ has to be computed in order to obtain an uncertainty estimate for time *t*. Because *z*_*t*_ is furthermore continuous, a finite number of samples is randomly drawn from the sensor models in order to compute the expected value *E*_*z*_*t*__. Update Eqs (3) and (4) (or rather, their sampling-based particle filter implementations) can then be applied to approximate the expected distribution after having executed action *u*_*t*_.

For measuring the uncertainty of the state, we only consider the positional uncertainty of the source and ignore the robot’s head rotation *x*_*t*;*r*_. We discretize the distribution over positions by a histogram where the probability of each bin is calculated from the number of particles located inside the bin. For this, it is essential to transform positions into a Cartesian representation first because the polar representation used by the particle filter has the disadvantage that the resulting histogram bins would not cover the same amount of area (when dividing both, angle and distance, linearly), which would distort the uncertainty estimate. Let $x_{t}^{\left(i\right)}$ denote the discretized position expressed in Cartesian coordinates corresponding to the *i*-th histogram bin. For this discretized distribution, the entropy *H* is given by

$$\begin{array}{c} H\left(x_{t}|z_{0:t},u_{0:t}\right)=-\sum_{i}P\left(x_{t}^{\left(i\right)}|z_{0:t},u_{0:t}\right)\log P\left(x_{t}^{\left(i\right)}|z_{0:t},u_{0:t}\right). \end{array}$$ (8)

Because the entropy for time *t* − 1 is constant with respect to *u*_*t*_ in Eq (7), it can be ignored for the optimization problem, and the maximization of the expected IG becomes a minimization of the expected entropy with

$$\begin{array}{c} \underset{u_{t}}{arg min}\left(E_{z_{t}}\left[H\left(x_{t}|z_{0:t},u_{0:t}\right)\right]\right). \end{array}$$ (9)

It is not possible to calculate the expected uncertainty for every realizable action *u*_*t*_ because all parameters (angle head rotation, angle robot rotation and distance of robot translation) are continuous. Thus, in order to choose the most informative action in practice, actions are sampled randomly. Moreover, in order to avoid situations in which all or most sampled actions coincidentally lead to similar target states, we ensure that important target positions are represented in the set of simulated actions at least once. For this, we divide each of the three components constituting an action in our system into subintervals and randomly sample at least one action within each combination of these intervals. Because the range of values of these parameters is continuous, the number of intervals and the number of simulated actions has to be decided based on the particular application. In the following, we use six intervals for the head direction, six intervals for the rotation of the robot and four intervals for translation distance (between 0.3m and 2.0m), which corresponds to 144 different actions. Once the expected IG has been computed for every action, the action resulting in the lowest expected uncertainty is executed by the robot.

While the calculation of the IG requires additional computational resources, it minimizes the number of actions the mobile robot has to execute in order to achieve accurate position estimates. Furthermore, in order to save computation time, it is always possible to use a reduced particle set for the calculation of IG. While this can potentially lead to worse estimates of the entropy of the resulting particle distribution, in most cases the accuracy is still sufficient. The main advantage of integrating movements of the system itself into our source localization system is that it allows integrating multiple consecutive measurements from different positions, which results in better estimates. In particular, this enables the robot to estimate the distance of the source, which is very hard to achieve in vision with a single measurement, and is to our knowledge practically impossible in audition.

## Results

In this section, we evaluate the performance of the proposed system and investigate whether the information gain procedure is an efficient solution to the action selection problem for source localization. We divide the presentation of our results in two subsections: In the first section, we present results of the proposed system in a simulation environment while, in the second section, we present results of the system running on the robot. The evaluation in the simulated environment mainly serves as proof of concept. The simulation environment allows for an easier and more thorough evaluation and quantification of the system’s reactions to different situations because no additional hardware-based tracking of the robot and the source is needed to compare estimated and actual positions. In contrast, experiments utilizing the robot are the only way to ensure that the proposed system is able to handle real-world applications.

### Simulation Experiments

At first, an example run of the system is presented in order to demonstrate the behavior of the system and to show the main principles of our approach. Second, quantitative results are presented, including an evaluation of the average estimation error and an analysis of the entropy of the PDF estimate.

To simulate auditory measurements, we are using test files which were recorded by the robot under real-world conditions. These test files were tagged by direction and were selected randomly while ensuring that the selected test file is appropriate for the respective relative position of the source. Since it is difficult to record realistic camera images for all possible states, we simulate visual measurements by generating them artificially. This is accomplished by generating feature vectors which resemble those resulting from the template matching and logistic regression approach described above. We approximate these responses by a mixture model consisting of a Gaussian for the bounding box of the source and a uniform distribution representing noise. The parameters of this model are fitted to a dataset annotated with the true positions and sizes of the source inside the image.

**Fig 5:**
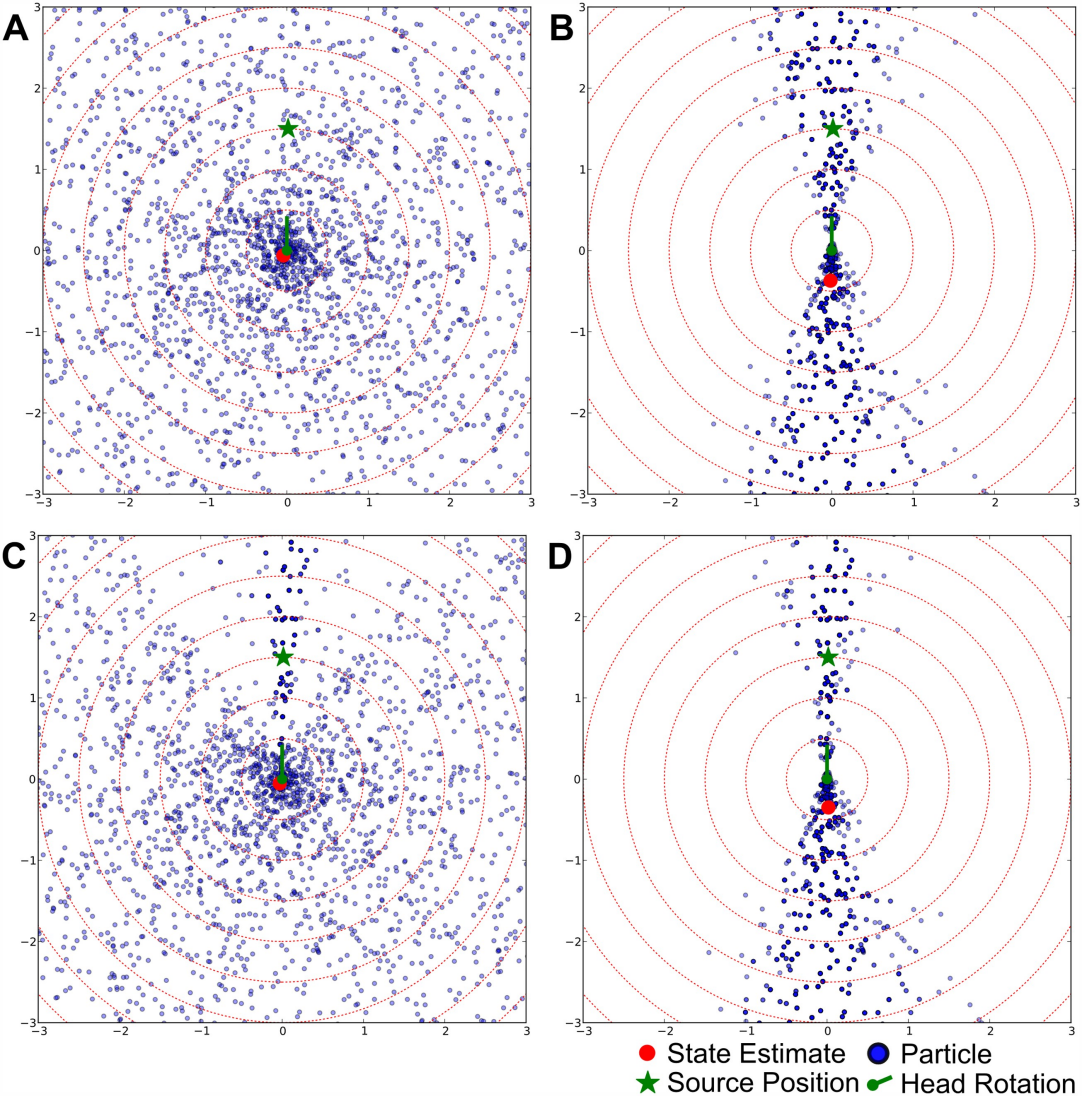
Initial State And Updates. These figures show the initial particle distribution in a robot-centric coordinate system whose origin corresponds to the center of the robot (indicated by a green dot and a green line which illustrates that the robot’s head is rotated toward its front) while the x- and y-axis correspond to the left/right and front/back of the robot, respectively. Particles are depicted as blue dots while the source’s actual position is indicated by a green star. The mean of the particle distribution is symbolized by a red dot. We chose to include it in these figures because we use it as a state estimate when calculating distances to the actual position of the source. **A) After initialization** all particles are distributed uniformly in the state space, which implies that the system does not have any information about the location of the source, which is located in front of the robot. **B) The initial auditory correction update** eliminates all particles located to the left and right of the robot because they are not compatible with the measurement. The width of the cone around the “true position” directly corresponds to the standard deviation parameter of the Gaussians in the auditory sensor model. This correction update is also a good example for the front-back confusion in audition: Based on the ITD-measurement, the system cannot distinguish whether the source is in front of or behind it, and thus treats corresponding particles behind and in front of it the same way. **C) The initial visual correction update** shows the characteristic properties of the visual modality within our system: Visual measurements have a high spatial precision but particles corresponding to positions outside the field of view of the camera are not updated because the measurement contains no information about these positions. **D) The combination of both sensory updates** shows the interaction of the modalities: The visual modality is able to increase the precision of the estimate based on the auditory measurement but provides no information about locations outside the field of view.

Fig 5A to 5D show the effects of the measurement updates for the individual modalities. In Fig 5A, the initial uniform particle distribution is shown, while Fig 5B to 5D illustrate the updates of the particle distribution for auditory and visual measurements and their combination. These figures illustrate an interesting synergy of modality-specific properties in our system: While auditory measurements are effective for reducing the number of possible hypotheses about the position of the source by a single measurement (auditory measurements update all particles at once), visual measurements offer a high spatial precision but a small field of view and thus can be used by the system to improve the position estimate based on the auditory measurement.

**Fig 6:**
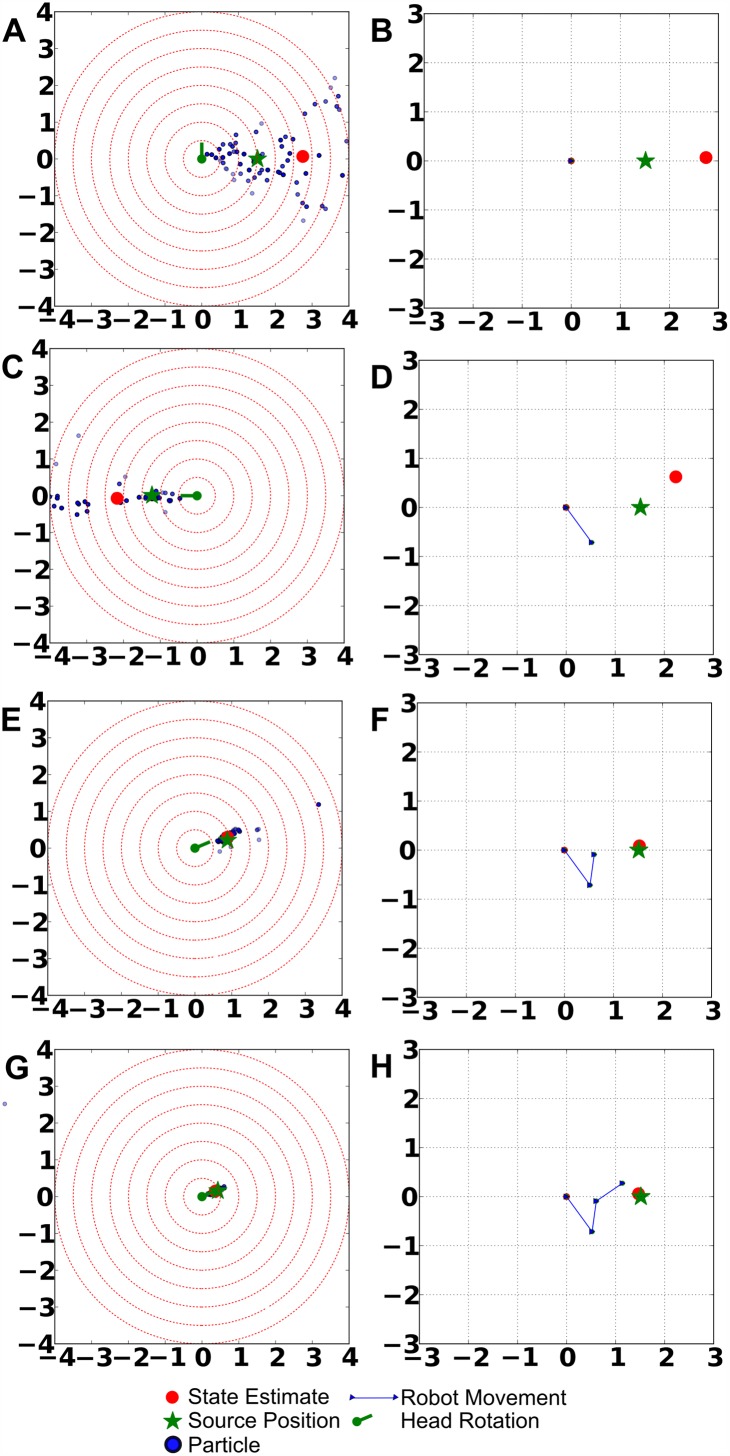
Example run of the system. The figures in the top row show the position of the simulated robot, the source, and the actions performed by the robot in a room-centric/absolute coordinate system, while the figures in the bottom row show the current particle distribution in the robot-centric coordinate system. We include both types of plots, because it is easier to analyze the particle distribution in a robot-centric coordinate system while the depiction of robot movements is only possible in an absolute reference system. A) and B): Initial state after the first measurement. C) and D): State after first movement and subsequent measurements. E) and F): State after the second movement. G) and H): State after the third movement.

To exemplify the system’s behavior, an example run of the system is shown in Fig 6A to 6H. These figures illustrate that, after initialization, the system utilizes audition to reduce the set of possible positions, exploiting the fact that audition can remove many hypotheses by a single measurement. As soon as the ambiguity of the particle distribution is sufficiently reduced, the robot turns its head towards the source for a more precise localization utilizing the visual modality. Moreover, these figures show a distinctive property of the proposed system: In order to improve the localization estimate, the simulated robot tends to approach the source, which can be explained by the fact that the auditory measurements are more precise if the distance to the source is small.

**Fig 7:**
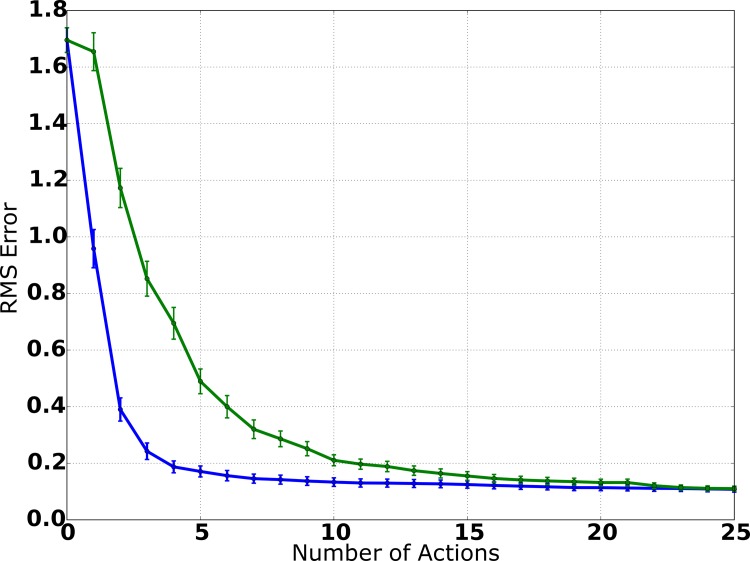
Average RMS error (simulation). Utilizing the IG procedure (blue curve), the average RMS error decreases monotonically with the number of actions performed by the system and only few actions are needed for a precise estimate. With random movements (green curve), localization accuracy is considerably worse, indicating that the IG procedure is an efficient solution for the selection of actions.

In Fig 7A, the average error of the position estimate is shown as a function of the number of actions. To measure the accuracy of the estimates, we calculate the root mean square (RMS) error by $err=abs(\text{x}-\hat{\text{x}})$ where **x** denotes the actual location of the source and $\hat{\text{x}}$ denotes the system’s position estimate. For this, **x** and $\hat{\text{x}}$ are both represented in Cartesian coordinates because the resulting values then directly correspond to the Euclidean distance between the system’s estimate and the actual position of the source, which makes interpretation easier. Fig 7A clearly shows that the system is able to localize the source with high accuracy: Initially (after the first measurement), the position estimate is still ambiguous, which is explained by the fact that the system has little information about the distance of the source, because the individual measurements of both modalities only provide information about the relative direction of the source. After the second action and consecutive measurements, the average RMS error is already below 0.4m and, after 4 actions, it is already smaller than 0.2m. This is a good result considering that the spatial precision of the measurements (particularly audition) is not very high and that the additive Gaussian noise for the robot’s translation movements is modeled with a standard deviation of 0.08m in the motion model. In summary, the system is able to produce an accurate position estimate and only needs few actions and consecutive measurements to accurately estimate the source position.

In order to evaluate whether the IG procedure actually improves localization performance, we conducted an additional set of experiments, where actions were selected randomly by the system and the IG procedure was deactivated. The results are shown in Fig 7B. Comparing the average RMS error of information-driven source localization to the results of source localization utilizing random movements, it is noticeable that the IG procedure leads to a substantial improvement of localization performance: The initial error (after the first sensory measurements) is similar for both approaches because the system has not performed any actions yet and thus there is no difference between both. But when comparing the results of both experiments after the system has executed actions, it is obvious that, in each step, the localization accuracy is considerably worse if movements are selected randomly.

**Fig 8:**
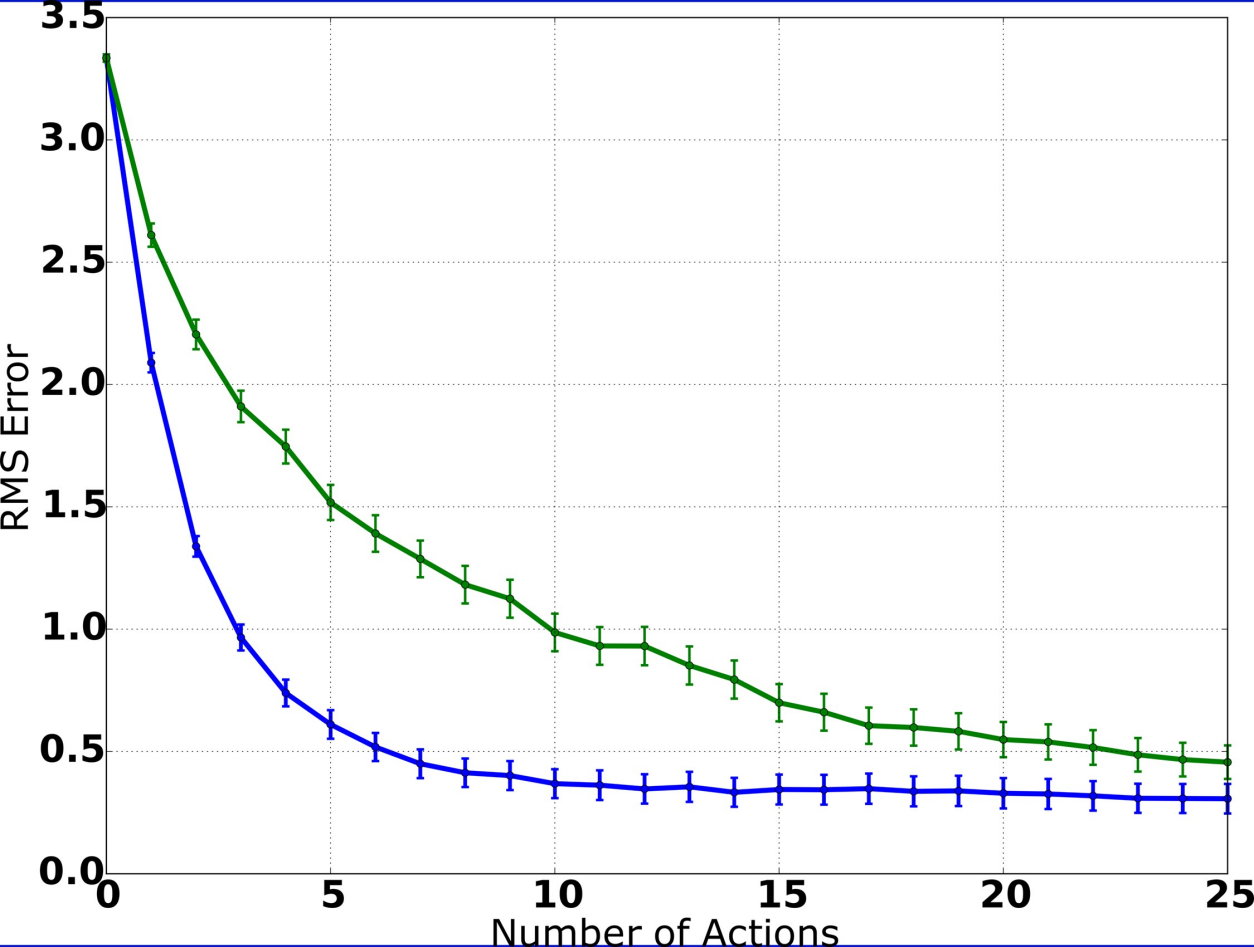
Entropy of the PDF estimate (simulation). When utilizing the IG procedure, the entropy of the PDF estimate decreases on average with each action, while the estimate of the source’s position gets more precise. While random movements also lead to a monotonic decrease of entropy, the comparison of the curves shows clearly that the IG procedure is reducing the remaining uncertainty more efficiently.

The IG procedure is based on the minimization of the expected entropy of the PDF estimate. Thus, in order to evaluate the action-selection process, we calculated the entropy averaged over all experiments as a function of the number of actions performed by the system and compared the results to the system variant utilizing random movements. The results are presented in Fig 8. The entropy of the estimated PDF decreases with each action and the number of actions needed to achieve an precise estimate is minimized. By interpreting entropy as a measure of uncertainty, these results show that the system is actively trying to reduce uncertainty with respect to the position estimate. The average entropy is clearly reduced with each action when utilizing the IG procedure, while random movements rarely lead to drastic reductions of entropy and the impact of each action is smaller in general.

**Fig 9:**
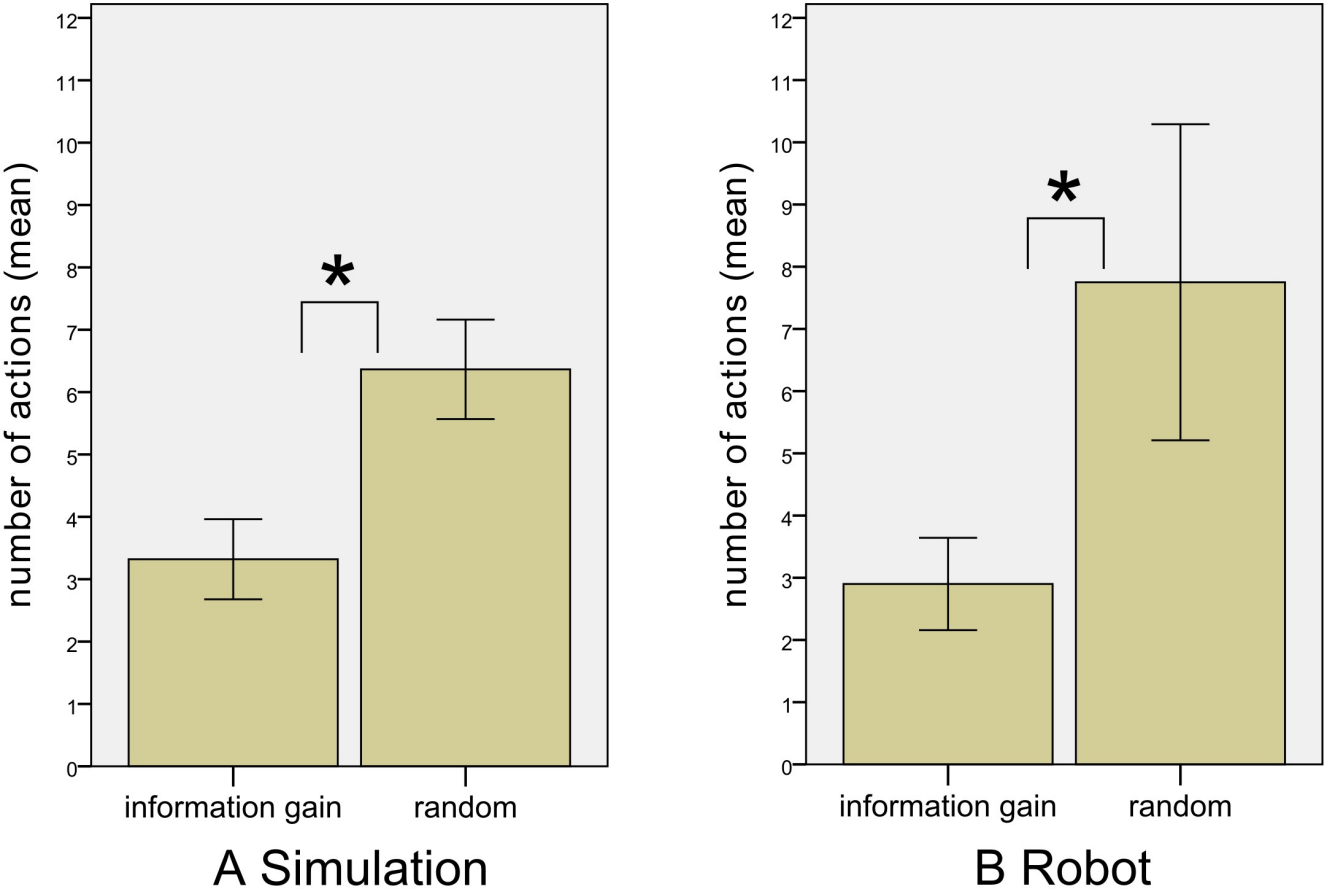
Localization performance. The result show a significantly lower number of actions required to reach a criterion RMS error < 30*cm* in both A) simulation and B) robot experiments. Errors bars show confidence intervals (.95).

Moreover, the number of actions required to reach a criterion RMS error of less than 30cm serves as an indicator of localization performance. As shown in Fig 9A, the IG procedure requires fewer actions to reach the criterion in comparison to randomly selected movements. This impression from descriptive statistics is confirmed by a Mann-Whitney test (*α* = .5) which indicates a significantly lower number of actions using the IG procedure (*Mdn* = 2) compared to random selection of movements (*Mdn* = 5), *U* = −8.67, *p* <.001.

### Robot Experiments

In this section, we present results of the system’s source localization capabilities under more realistic conditions implemented on a robot that is able to move freely inside an empty room. For now, we are using an empty room in order to abstract from obstacle detection and avoidance. We used cardboard to separate an area of 5.5m × 4.5m from the rest of the 7m × 6m room in order to provide a relatively uniform background for the template matching algorithm and to protect the robot from driving into walls or other objects due to technical failures. To continuously determine the actual position of the robot and its relative position to the source, we use the robot’s integrated dead reckoning. Apart from the fact that the data presented in this section is collected by the actual robot system, we are using the same procedure as above.

We kept the target elevation consistent (1.2m above the floor) between experiments because our system is currently designed for azimuthal localization only and we wanted to minimize sources of error. The initial relative position between the robot and the source was measured manually. As the source is assumed to be static in our setup, its position relative to the robot only has to be measured once before each experiment. In order to minimize measurement errors, we chose to select only initial positions for which the source was located on one of the main axes of the robot-centric coordinate system. That is, it was located either directly in front of, behind, to the left, or to the right of the robot to simplify the measurement of the initial position of the source relative to the robot. This simplification is justified because the simulation results show that the initial relative position of the source is mostly irrelevant for localization performance in our setup. The distance was varied randomly between -2m and 2m. We conducted a total of 20 experiment runs, which seemed to be a good compromise between expenditure of time and statistical interpretability.

Most natural sounds of interest for our application are relatively broadband and contain significant low-frequency energy. We thus used high-bandwidth noise patches as auditory stimuli which contain sufficient low-frequency components to make the auditory localization based on ITDs feasible. We use a cylindric object textured with a black and white pattern as a visual stimulus, which is relatively easy to detect utilizing the template-matching-based object detection approach. In order to achieve better performance during visual object detection, we created a set of 112 templates for different positions, robot orientations, and distances.

**Fig 10:**
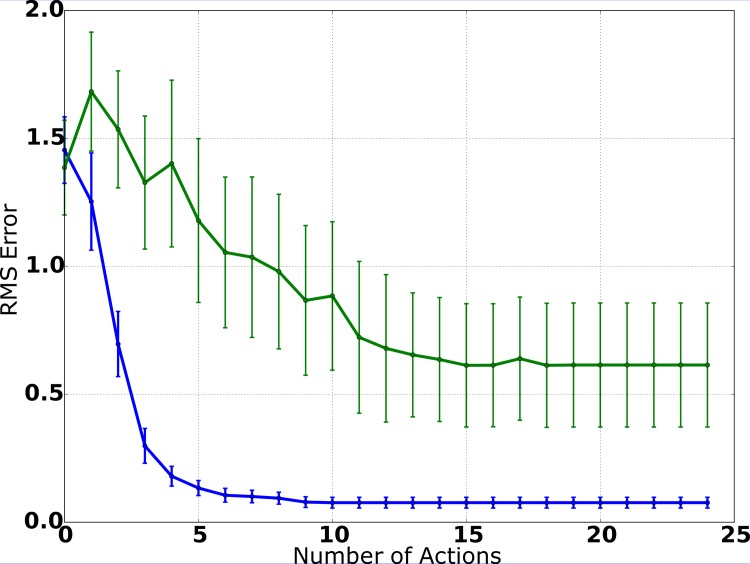
Average RMS error (robot experiments). Confirming the results of the simulation experiments (Fig 7), the average RMS error smoothly decreases with every action and few robot movements are needed to achieve a precise position estimate. When deactivating the IG procedure, localization performance gets considerably worse, even more so than in the simulation environment.

**Fig 11:**
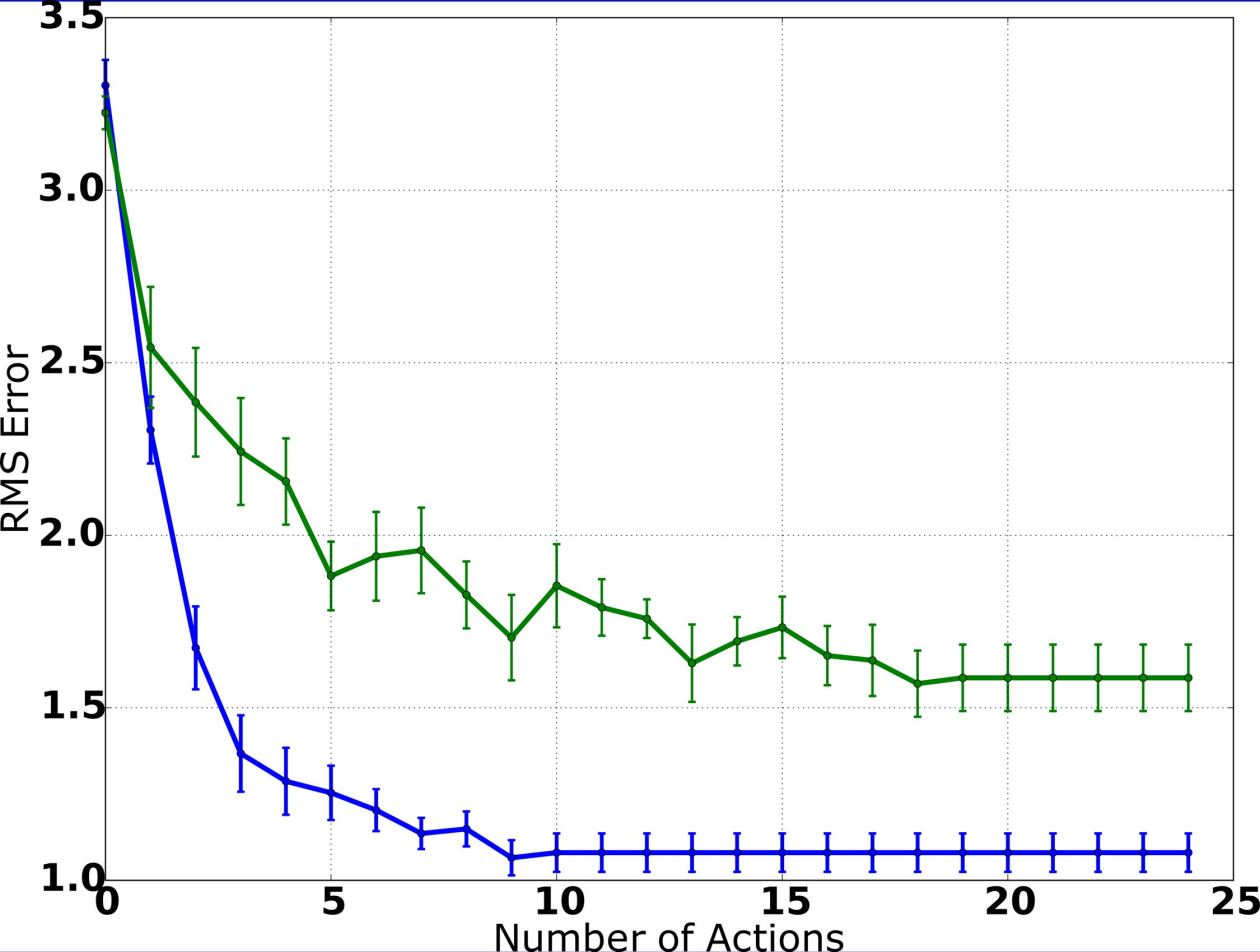
Entropy of the PDF estimate (robot experiments). The information-driven action selection process leads to a (roughly) exponential decrease of entropy. In comparison, random movements still lead to a mostly monotonic decrease of entropy, but at a slower rate and with a higher minimum entropy than in the information-driven approach.

Figs 10 and 11 show the results of the robot experiments. In Fig 10A, the average RMS error is plotted as a function of the number of actions, averaged over all experiments. The initial error after the first measurement is similar to the simulation results, showing that sensory measurements are of similar quality in simulation and in the robot scenario. After the first movement and subsequent visual and auditory measurements, the average RMS error is still 1.25m, which can be explained by the fact that, after one movement and two measurements, the system has limited distance information. After the second movement, the average error already decreases to less than 0.7m, and it continues to decrease to ca. 0.3m after the third movement. After the fourth movement, the average RMS error is below 0.2m, which already is smaller than the diameter of the source. Overall, the plot shows a smooth monotonic decrease of the average error, marginally worse than the results produced in the simulation environment. This is especially an interesting result because auditory measurement precision is worse than during simulation due to highly reverberant signals and the relatively simple template-matching-based vision system described above, which is principally prone to noise and illumination changes. Moreover, the combination of possible measurement errors for the initial position and the radius of the source also might affect performance negatively.

In Fig 10B, we compare the system’s performance to the performance of a system variant, in which actions are selected randomly in each step and the IG procedure is deactivated. The results are also averaged over 20 experiment runs to ensure comparability. While the average error smoothly decreases when utilizing the IG procedure, random movements lead to a slower decrease and never reach the performance of the information-driven approach. After 5 steps, the average RMS error is still greater than 1.0m and, even after 10 steps, it is just slightly under 1.0m. Moreover, the average error never gets smaller than 0.6m, probably due to the fact that (in contrast to the information-driven variant) the system does not systematically approach the source and thus cannot increase the spatial precision of the estimate because of the limited precision of auditory measurements for high distances.

In Fig 11A, the entropy of the PDF estimate is plotted as a function of the number of actions, averaged over all experiments. Again, these are similar to the results in the simulation environment (Fig 8A), as the entropy of the PDF estimate decreases monotonically with each action. When comparing these results to those of the system variant utilizing random movements (Fig 11B), it is obvious that the IG mechanism is successful in actively reducing uncertainty with respect to the state. The utilization of the IG procedure leads to considerably better results when compared to the results of the system variant utilizing random movements: In each step, the average entropy is considerably lower, while the average RMS error is substantially smaller.

A Mann-Whitney test (*α* = .05) supports descriptive statistics by indicating that the number of actions required to achieve an RMS-Error of less than 0.3m is significantly lower using the IG procedure (*Mdn* = 3) compared with random selection of movements (*Mdn* = 6), *U* = −3.40, *p* = .001. This effect can also be seen in Fig 9B.

## Discussion

We were able to show that the proposed system can use a sensorimotor audio-visual strategy to localize a source with high efficiency and accuracy. This was demonstrated both in a simulated environment and under real-world conditions using a robot. Each action reduces the uncertainty about the current state and, as a result, the system is able to accurately estimate azimuth and distance of the source despite substantial limitations in our setup, like simplified unisensory processing and “enforced” front-back-mixups in audition. As intended, only few actions are needed in order to achieve accurate position estimates, which shows that the IG mechanism is an efficient solution for selecting actions.

Furthermore, we showed that auditory and visual processing complement each other and that the combination of both modalities allows for the disambiguation of competing hypotheses. For example, we observed interesting multisensory interactions: While audition has a full 360 deg range (front/back ambiguities aside) and updates all particles independent of their direction, the visual modality has only a restricted field of view but therein a very high spatial precision. Consequently, the system utilizes audition to reduce the number of hypotheses very quickly and then uses vision to achieve more accurate estimates (see Fig 6 for comparison). We could show that localization performance is significantly reduced when replacing the IG mechanism with randomly chosen movements.

Moreover, we showed that the system is able to estimate the distance of a source without explicit distance measurements. This is due to the particle filter combining multiple angle measurements from different positions into a distance estimate. In addition, the system is able to produce accurate estimates of the source’s position without any visual measurements. This makes our approach a suitable and cost-efficient alternative to auditory source localization approaches utilizing expensive microphone arrays, at least for applications where measurements for different positions are available.

Because movements are an integral part of the system architecture, the main purpose of the proposed source detection and localization algorithm is the application in mobile robotics. But it is also applicable to stationary systems, which are able to perform rotations, making it suitable for applications such as automatic camera control systems for video conferences. An interesting area of application for the proposed system is audio-visual detection and localization of a human speaker in noisy and cluttered environments. This is, for example, an essential feature for the camera control systems mentioned above, and for rescue robotics where the localization of victims and survivors by mobile robots in cluttered environments is an important area of research. In rescue robotics, the utilization of a mobile robot with auditory and visual sensors is particularly useful because the robot’s mobility allows for free movement and thus can be used in complex environments assuming that a robust mechanism for obstacle avoidance is available. Note that our system can cope with situations where the person of interest is not visible from certain positions.

In preliminary experiments, we use a people detection approach based on a multiscale pedestrian detector [42]. The combination of this detector with our information-driven multisensory approach shows very promising preliminary results for this application.

While the system is currently designed to work with only a single static source, it is easily expandable to handle multiple dynamic sources by including an arbitrary source separation mechanism for the audio data (e.g., based on Onset and Offset positions [43]). After separating the sources, it is possible to apply the existing auditory source localization algorithm: For each source, ITDs have to be calculated using only those channels associated with the respective source as estimated in the segmentation stage. This would not require any significant additional computational costs because the number of channels within the cochleagram remains constant. In the future, we will extend the auditory processing to allow localization in the median plane utilizing filter characteristics of the artificial pinnae, which introduce position-dependent Head-Related-Transfer-Functions [44].
