# Correction: Information-Driven Active Audio-Visual Source Localization

**DOI:** 10.1371/journal.pone.0177588

**Published:** 2017-05-08

**Authors:** The PLOS ONE Staff

## Notice of Republication

This article was republished on April 3, 2017, due to incorrect figures that were published in the web version of the article. The publisher apologizes for the errors.
